# Joy

**Published:** 1889-11-16

**Authors:** 


The birds as they sing, the lamb as it skips, the angels as they watch the sinners return to God; all are moved with deepest joy." And, therefore, since religion is merely obedience to the laws of God, we may be sure He who made man and gave religion will expect to find joy in every religious heart.

On the other hand, what misery and discontent a grumbling melancholy nature brings on itself and all around it. The Jews were always murmuring in the wilderness. First they wanted one thing, and then another; everything was wrong, they were constantly finding fault, and though God gave them what they asked for, punishment came with it, and they were destroyed of the destroyer. So it is with the individual, a murmuring, joyless life will be fatal to growth in grace.

But we should dwell on this truth that for a Christian all his joy must be "in the Lord." All delights which are silly or sinful, all frivolous pleasures are only destruction of true joy. Those amusements over which we cannot say a prayer, those companionships where Jesus cannot be invited as a fellow guest, are surely not joy in the Lord. The real groundwork of joy is contentment with the will of God, a hearty acceptance of all He sends us, for if there is a difference between the two wills what misery and chafing sets in! Discontent, fretting, jealousy of others, anxiety, are all of our own making. If we only give ourselves up entirely to God, and are influenced by His love, our failures, our disappointments, our sufferings will be turned to blessings, our bodies will joyfully minister to the wants of others, and our hearts will sing one long *Magnificat*, "My soul doth magnify the Lord and my spirit hath rejoiced in God my Saviour."

